# Basement-Membrane-Related Gene Signature Predicts Prognosis in WHO Grade II/III Gliomas

**DOI:** 10.3390/genes13101810

**Published:** 2022-10-07

**Authors:** Zhaogang Zhang, Guichuan Lai, Lingling Sun

**Affiliations:** 1 Department of Radiology, The Fourth Affiliated Hospital of China Medical University, Shenyang 110032, China; 2 Department of Epidemiology and Health Statistics, School of Public Health, Chongqing Medical University, Chongqing 400016, China

**Keywords:** basement membrane, gliomas, prognostic-related gene, nomogram

## Abstract

Gliomas that are classified as grade II or grade III lesions by the World Health Organization (WHO) are highly aggressive, and some may develop into glioblastomas within a short period, thus portending the conferral of a poor prognosis for patients. Previous studies have implicated basement membrane (BM)-related genes in glioma development. In this study, we constructed a prognostic model for WHO grade II/III gliomas in accordance with the risk scores of BM-related genes. Differentially expressed genes (DEGs) in the glioma samples relative to normal samples were screened from the GEO database, and five prognostically relevant BM-related genes, including *NELL2*, *UNC5A*, *TNC*, *CSPG4*, and *SMOC1*, were selected using Cox regression analyses for the risk score model. The median risk score was calculated, based on which high- and low-risk groups of patients were generated. The clinical information, pathological information, and risk group were combined to establish a prognostic nomogram. Both the nomogram and risk score model performed well in the independent CGGA cohort. Gene set enrichment analysis (GSEA) and immune profile, drug sensitivity, and tumor mutation burden (TMB) analyses were performed in the two risk groups. A significant enrichment of ‘Autophagy–other’, ‘Collecting duct acid secretion’, ‘Glycosphingolipid biosynthesis–lacto and neolacto series’, ‘Valine, leucine, and isoleucine degradation’, ‘Vibrio cholerae infection’, and other pathways were observed for patients with high risk. In addition, higher proportions of monocytes and resting CD4 memory T cells were observed in the low- and high-risk groups, respectively. In conclusion, the BM-related gene risk score model can guide the clinical management of WHO grade II and III gliomas.

## 1. Introduction

Gliomas represent the most commonly diagnosed primary cancer of the brain in grown-ups [1], and 45% of glioma cases are classified as World Health Organization (WHO) grade II or III [2]. Due to their highly aggressive nature and anatomical location, gliomas are difficult to surgically remove. This affects patient prognosis, and some cases progress to glioblastoma within a few months [3,4]. Therefore, it is important to screen for genes associated with the prognosis of gliomas, which can help predict patient survival and facilitate early intervention in high-risk patients to improve their prognosis.

Basement membranes (BMs) are fine, nanoscale, and flexible extracellular matrix structures [5,6] involved in maintaining tissue separation and barriers, cell adhesion, and cell migration [7,8,9,10]. Studies show that the BMs are a major determinant of the invasiveness and metastatic potential of cancer cells [11,12,13], and vascular BM components are involved in regulating tumor angiogenesis [14]. A recent study revealed genes associated with basement membranes [5], providing new insights into the association of basement membranes with disease and, in particular, their important role in the development and prognosis of gliomas, as suggested by previous studies. These genes include the A Disintegrin and Metalloproteinase (ADAM) family, a superfamily of zinc-dependent metalloproteinases, some members of which are considered to be diagnostic and prognostic markers for central nervous system (CNS) tumors. Qu et al. [15] showed that the mRNA and protein expression levels of ADAM10 were malignancy-dependent in gliomas and that they may be associated with the formation of peritumoral edema. Furthermore, Zheng et al. [16] found that *ADAM17* played a role in promoting glioma proliferation and invasion. *LOXL1*, which belongs to the LOX family, has been shown to protect glioma cells against apoptosis and promote glioma development [17]. A recent study also demonstrated that *ITGB2* can be a new predictor of glioma prognosis and immunotherapy response [18]. In addition, related studies have shown correlations between genes, such as *ROBO4* [19] and *DDR1* [20], and glioma prognosis.

Nevertheless, the relationship between BM-related genes and glioma remains unclear, and the identification of new biomarkers is essential for clinical decision making. Thus, we aimed to explore the predictive value of BM-related genes for overall survival (OS) in WHO grade II and III gliomas and to identify a novel biomarker.

## 2. Materials and Methods

### 2.1. Data Extraction

**Figure 1:**
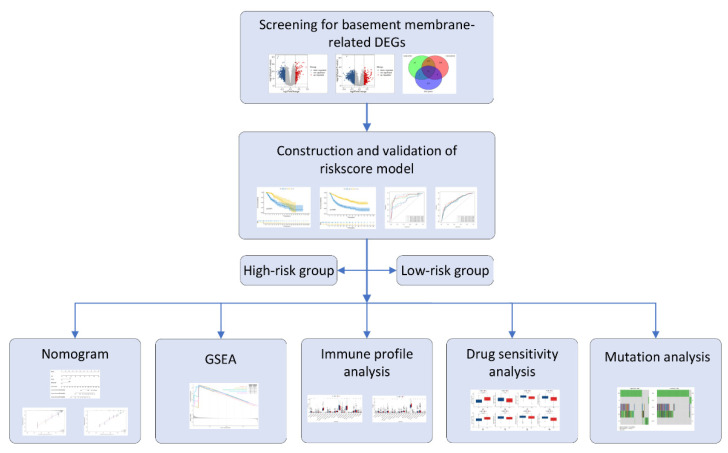
Workflow of the BM-related gene signature construction.

The GSE4290 [21] and GSE68848 [22] datasets were retrieved from the GEO (http://www.ncbi.nlm.nih.gov/geo/, accessed on 22 June 2022) database. The gene expression, tumor mutation burden (TMB), and clinical information of patients with low-grade glioma (LGGs) were obtained from TCGA database (https://portal.gdc.cancer.gov/, accessed on 6 June 2022). The mRNAseq_325 [23,24,25] and mRNAseq_693 [23,26,27] datasets, which included the gene expression and clinical data of two groups of glioma patients, were retrieved from the CGGA (http://www.cgga.org.cn/, accessed on 7 June 2022). The WHO grade II/III cohorts from the TCGA and CGGA were selected for the follow-up analysis, and cases with unknown OS and OS < 30 days were not included. BM-related genes were obtained from previously published articles [5]. The analytical procedure is outlined in Figure 1.

### 2.2. Initial Screening of BM-Related Genes

The differentially expressed genes (DEGs) of the WHO grade II/III gliomas versus control samples in the GSE4290 and GSE68848 datasets were screened using the “limma” program in R. The cutoffs were |log2fold change (FC)| > 1.5 and *p*-value (adjusted by) <0.05. The intersecting DEGs and BM-related genes were further selected for subsequent analysis. In addition, based on the optimal cutoff, Kaplan–Meier (KM) survival analysis was conducted on the intersecting genes to investigate their relationship with prognosis. The optimal cutoff value was obtained based on the “surv_cutpoint” function of the R package.

### 2.3. Development of the Risk Score Model

The prognostically significant BM-related genes were screened from the TCGA cohort (training set) using univariable and multivariable Cox regression analyses. Risk scores were calculated and a model was constructed. The median risk score was then calculated to demarcate the low- and high-risk patients. To validate this model, patients in the CGGA cohort were subjected to risk score calculation, and risk groups were similarly generated.

### 2.4. Establishment and Verification of a Predictive Nomogram

The independent prognostic value of the risk group was determined by adjusting for patient age, gender, and WHO grade. A predictive nomogram was then constructed by combining the risk group with the clinical and pathological information, and its predictive efficacy was appraised by using time-dependent C-index plots and calibration plots.

### 2.5. Gene Set Enrichment Analysis (GSEA)

We performed GSEA on both TCGA cohort risk groups to identify the differential gene pathways.

### 2.6. Immune Infiltration Analysis

The relative proportions of 22 immune cells were calculated using the CIBERSORT algorithm. The immune and stromal scores were calculated using the ESTIMATE algorithm, and, finally, the TIDE scores of the patients were calculated using the TIDE database. Differences between the different risk groups were visualized using violin plots, and all analyses were performed in both the TCGA and CGGA cohorts.

### 2.7. Drug Sensitivity Analysis

The sensitivity of the patients in both risk groups to axitinib, cisplatin, dasatinib, and pazopanib was compared using the “pRRophetic” R package.

### 2.8. Mutation Analysis

Based on the TCGA TMB data, the mutation frequencies of *IDH1*, *IDH2*, *ATRX*, *TP53*, and *EGFR* were compared between the two risk groups. 

### 2.9. Statistical Analysis

All RNA-seq data were converted into transcripts per million (TPM) and normalized by log(x + 1). Batch correction was performed using the “sva” package, and batch effects were assessed by using principal component analysis (PCA). The OS curves were generated with the Kaplan–Meier method and subjected to a log-rank test. The model’s discriminatory power was assessed using ROC curves, AUC values or time-dependent C-index plots and the calibration thereof were assessed using calibration curves.

R 4.1.3 software (R Development Core Team, Auckland, New Zealand) was utilized for the analysis of the statistical data, and a *p*-value of <0.05 was deemed to signify statistical significance.

## 3. Results

### 3.1. Initial Selection of BM-Related Genes

**Figure 2:**
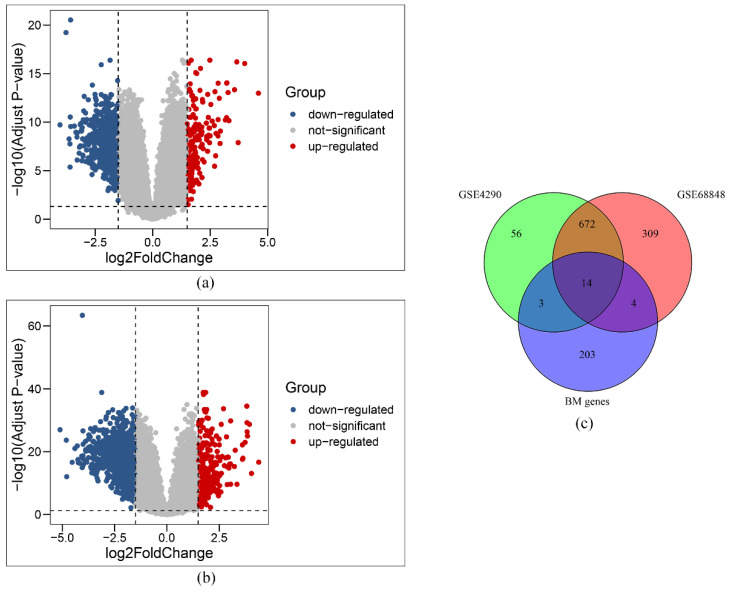
Identification of DEGs in BM-related genes. Volcano plots showing the DEGs in GSE4290 (**a**) and GSE68848 (**b**). VENN plot showing the intersection of DEGs and BM-related genes (**c**).

A total of 745 and 999 DEGs, respectively, were identified in the GSE4290 and GSE68848 datasets. The volcano plots are shown in Figure 2a and Figure 2b. As shown in the VENN plot in Figure 2c, 14 intersecting BM-related genes and DEGs were identified and used for downstream analyses. Survival analysis showed that 12 genes (*SPARC*, *CCDC80*, *BCAN*, *VCAN*, *SPOCK3*, *NELL2*, *UNC5A*, *TNC*, *ADAMTS9*, *CSPG4*, *SMOC1*, and *ADAMTS19*) were related to prognosis, whereas two genes (*UNC5D*, *TENM2*) were not (Figure S1).

### 3.2. Construction and Verification of the Risk Score Model

The training and validation sets included 460 and 589 patients, respectively. After univariable Cox regression analysis, we screened eight genes (*p* < 0.05) that also proved to be prognostically relevant in the survival analysis (*BCAN*, *NELL2*, *UNC5A*, *TNC*, *ADAMTS9*, *CSPG4*, *SMOC1*, and *ADAMTS19*). Subsequently, we screened again using multivariate Cox regression analysis (*p* < 0.05) and, finally, constructed BM-related risk score models based on the genes *NELL2*, *UNC5A*, *TNC*, *CSPG4*, and *SMOC1*. Risk score = 0.27761 × Exp*_NELL2_* − 0.39196 × Exp*_UNC5A_* + 0.23357 × Exp*_TNC_* + 0.28250 × Exp*_CSPG4_* − 0.17451 × Exp*_SMOC1._*

**Figure 3:**
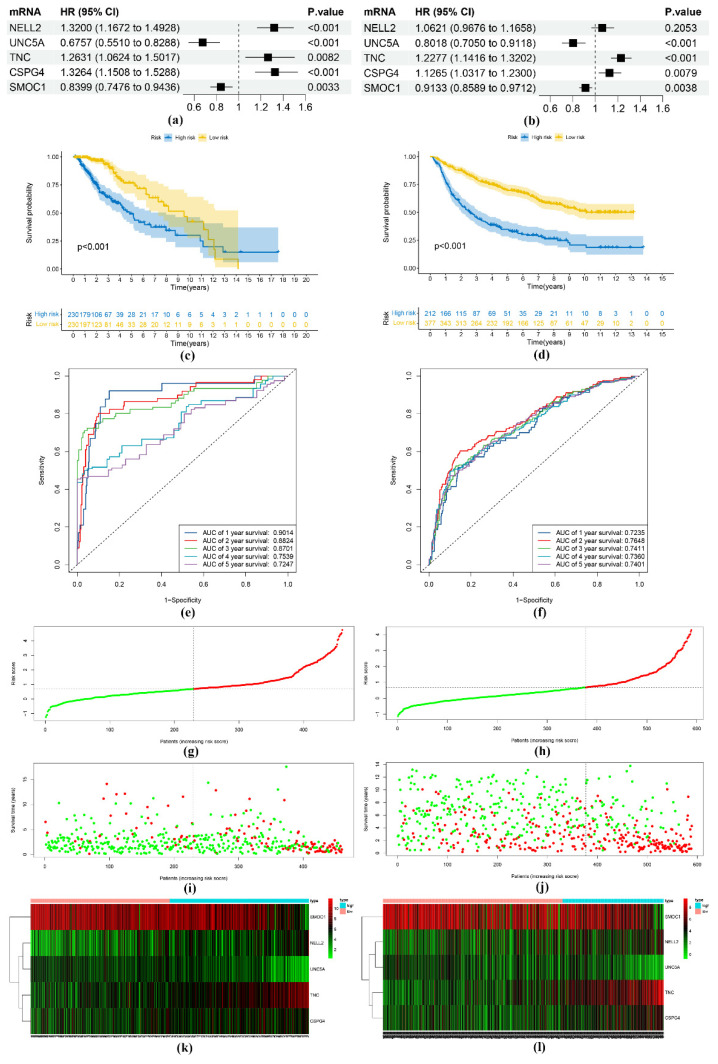
Establishment and verification of a risk score model. HR values of risk-score-model-related genes in the TCGA (**a**) and CGGA cohorts (**b**). Survival analysis of the risk score in the TCGA (**c**) and CGGA cohorts (**d**). ROC curves for 1-, 2-, 3-, 4-, and 5-year risk scores in the TCGA (**e**) and CGGA cohorts (**f**). Distribution of risk scores (**g**,**h**), patients’ survival (**i**,**j**), and heatmap of 5 BM-related genes (**k**,**l**) in the TCGA and CGGA cohorts, respectively. The green dots in the figures indicate survival, and the red dots indicate death.

In both the training and validation sets, the HR values of the genes *NELL2*, *TNC*, and *CSPG4* were >1, and the HR values of the genes *UNC5A* and *SMOC1* were <1 (Figure 3a,b). Furthermore, the low-risk group of patients exhibited longer survival compared to that of the high-risk group of patients (Figure 3c,d), and the ROC curves (Figure 3e,f) demonstrated that the risk scores in years 1, 2, 3, 4, and 5 had good predictability. The AUC values exceeded 0.8 for the first three years of the training set and 0.7 for the last five years of the validation set. Both sets exhibited a better OS for low-risk patients compared with that of their high-risk counterparts, as revealed by the risk score distribution (Figure 3g,h) and survival status (Figure 3i,j). A heatmap plotted based on the differential expression of the above five genes between the high- versus low-risk groups (Figure 3k,l) showed the same trend as the HR values of the genes.

### 3.3. Establishment and Verification of a Nomogram

**Figure 4:**
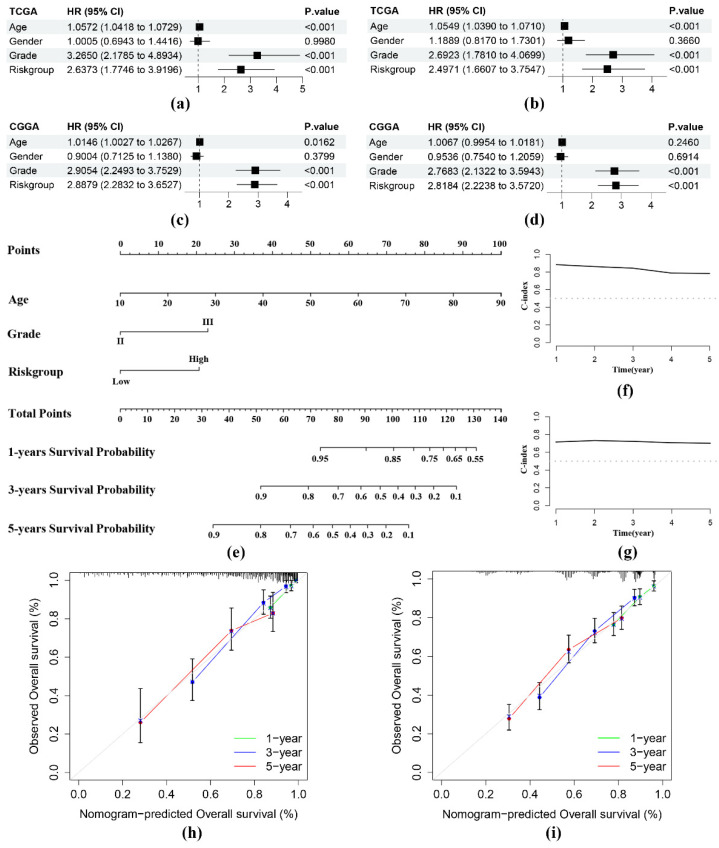
Evaluation of the predictive abilities of the nomogram. Univariable and multivariable Cox regression analyses of age, sex, grade, and risk group in the TCGA (**a**,**b**) and CGGA (**c**,**d**) cohorts. (**e**) Prediction of 1-, 3-, and 5-year OS in patients with WHO grade II/III gliomas by utilizing the nomogram. (**f**,**g**) Time-dependent C-index plots of the nomogram in both cohorts. (**h**,**i**) Calibration curves of the 1-, 3-, and 5-year OS in both cohorts for the nomogram.

We found the independent prognostic significance of the risk group in both the training and validation sets as per the regression analyses (Figure 4a–d). Therefore, we established a nomogram using the information on age, WHO grade, and risk group in the training set (Figure 4e). The time-dependent C-index plots at the fifth year remained approximately 0.8 in the training set (Figure 4f) and approximately 0.7 in the validation set (Figure 4g); these were performed consistently in both groups. The calibration curves of 1-, 3-, and 5-year OS (Figure 4h,i) showed the good calibration ability of the nomogram.

### 3.4. GSEA Findings

**Figure 5:**
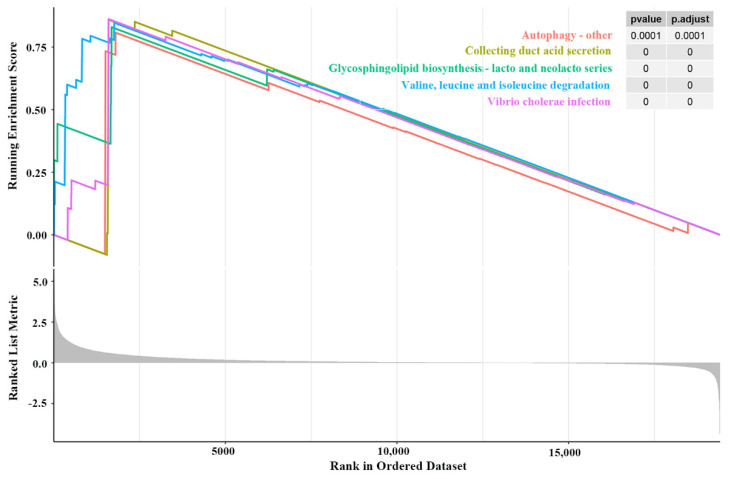
GSEA between the two risk groups in the TCGA cohort.

The ‘Autophagy–other’, ‘Collecting duct acid secretion’, ‘Glycosphingolipid biosynthesis–lacto and neolacto series’, ‘Valine, leucine, and isoleucine degradation’, ‘Vibrio cholerae infection’, and other pathways were notably enriched in the high-risk group (Figure 5). Some of these pathways have been implicated in the survival of glioma patients. Our findings suggest possible mechanisms underlying the worse survival outcomes of high-risk patients.

### 3.5. Immune Infiltration Analysis

**Figure 6:**
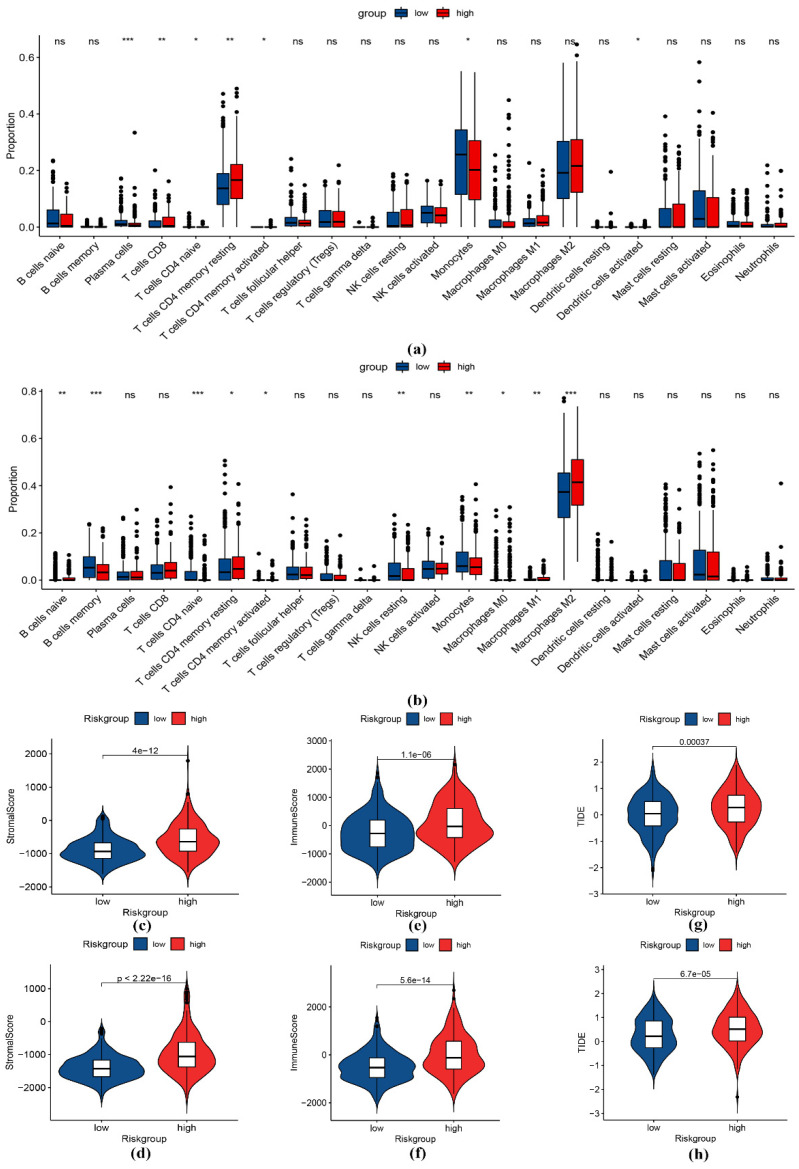
Immune profile analysis between the risk groups. Immune infiltration analysis in the TCGA (**a**) and CGGA (**b**) cohorts. Comparison of the stromal score (**c**), immune score (**e**), and TIDE score (**g**) in the TCGA cohort. Comparison of the stromal score (**d**), immune score (**f**), and TIDE score (**h**) in the CGGA cohort. * represents *p* < 0.05; ** represents *p* < 0.01; *** represents *p* < 0.001.

As shown in Figure 6a and 6b, the results from the TCGA and CGGA cohorts consistently showed that the distribution of naive CD4 T cells, resting memory CD4 T cells, activated resting memory CD4 T cells, and monocytes were significantly correlated with the prognosis of patients. Furthermore, monocytes had higher proportions in the group that was at low risk, and resting memory CD4 T cells had higher proportions in the group that was at high risk. In addition, the M2 macrophages were the predominant infiltrating immune cell population in the high-risk patients in both cohorts. In both cohorts, the high-risk group had a higher stroma score (Figure 6c,d) and immune score (Figure 6e,f), while the low-risk group had a lower TIDE score (Figure 6g,h) than the high-risk group, suggesting that the low-risk group might benefit more from immunotherapy.

### 3.6. Analysis of Patients’ Sensitivity to Selected Drugs

**Figure 7:**
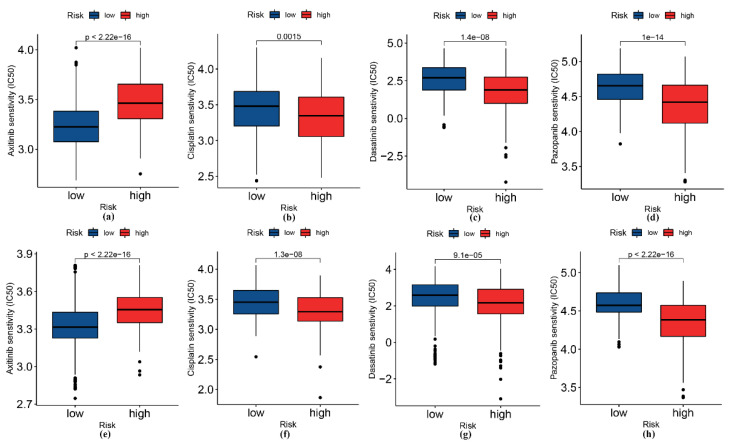
Sensitivity analysis between the high- and low-risk groups for four drugs: axitinib, cisplatin, dasatinib, and pazopanib. (**a**–**d**) TCGA cohort. (**e**–**h**) CGGA cohort.

The high-risk patients displayed better responses to cisplatin, dasatinib, and pazopanib, whereas the low-risk patients were more sensitive to axitinib (Figure 7).

### 3.7. Mutation Analysis

**Figure 8:**
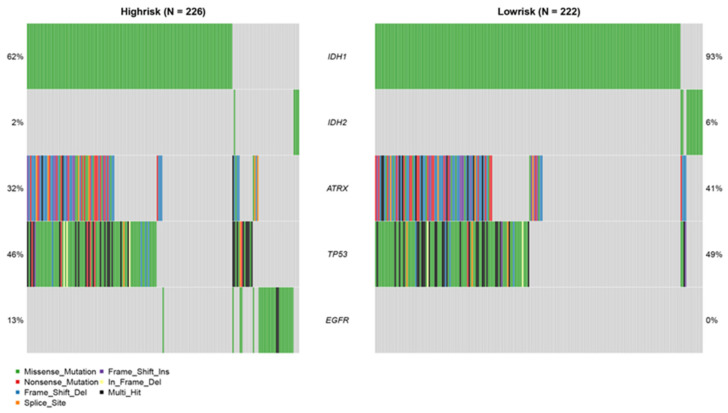
Mutation analysis in the TCGA cohort.

Higher frequencies of *IDH1/2* and *ATRX* mutations were detected in the low-risk patients, whereas patients classified as high-risk had more *EGFR* mutations. On the other hand, the frequency of *TP53* mutations was similar in both risk groups (Figure 8).

## 4. Discussion

We constructed a risk score model for WHO grade II/III gliomas based on BM-related genes, which we consider a novel candidate biomarker. This has not been reported in previous studies. In addition, we combined the risk group with the clinical and pathological information to develop a nomogram. Both the model and the nomogram were validated in the CGGA cohort. Furthermore, the patients demarcated by the median risk score also differed in terms of enriched pathways, immune profile, drug sensitivity, and TMB, which may help formulate personalized treatment regimens for glioma patients.

Moreover, exploring the proteins encoded by the identified genes helps to understand the findings. NELL2 (protein of the neural-tissue-specific epidermal growth factor-like repeat structural domain) [28] is a secreted glycoprotein that is expressed primarily in neural tissue and associated with neuronal differentiation. In recent years, it has been found to be associated with axonal development in hippocampal neurons. UNC5A [29,30], belonging to the netrin-1 receptor family, plays an important role in neuronal development and differentiation. It has been associated with the development of a variety of tumors, including through tumor suppression, and has also been shown to promote apoptosis independently of Netrin-1. TNC [31] is an extracellular matrix protein that is primarily associated with the regulation of cell adhesion, migration, and proliferation, and its sustained expression is associated with inflammation and a variety of tumors, including gliomas. Chondroitin sulfate proteoglycan 4 (CSPG4) [32,33] is a surface type I transmembrane core proteoglycan that is important in cell survival, migration, and angiogenesis and has been associated with the progression and metastasis of tumors, such as gliomas and soft tissue sarcomas. SMOC1 [34] is a calcium-dependent conformational glycoprotein and a true basement membrane component, which has been suggested to be a new cancer-related protein and has been shown to interact with TNC in vitro [35]. These protein products are important molecules that play a role in tumor development and even metastasis. Some of these proteins are associated in in vitro assays, and, at the protein level, we may hypothesize that these proteins and protein–protein interactions have a complex impact on the progression of gliomas and, thus, on patient prognosis, as we will explore further.

Previous studies indicated that monocytes can promote tumor growth and metastasis [36,37]. At the same time, they can play a role in preventing tumor development and spread [38]. Numerous studies are currently ongoing to uncover the mechanisms underlying the bidirectional role of monocytes. In our study, the immune infiltration analysis showed that monocytes were associated with a good prognosis, while the opposite was true for resting memory CD4 T cells, in line with previous studies [39]. In addition, previous studies have also revealed that the M2 macrophages confer dismal prognosis in gliomas [40] and other types of tumors [41]. The mechanistic roles of macrophages in cancer progression have been summarized elsewhere [42].

We have also explored the sensitivity of common chemotherapy drugs in different risk groups; this can provide valuable information for personalized treatment of patients. Notably, the potential of psychotropic drugs in the treatment of gliomas has emerged in recent years with the further development of drug repositioning, which may lead to new breakthroughs in the treatment of gliomas [43,44,45].

The *IDH* mutation status was involved in the 2016 guidelines of the WHO Classification of Tumors of the Central Nervous System for diagnosing and classifying gliomas [46]. In fact, the presence of *IDH* variations in glioma patients correlates with longer survival compared to that of patients lacking mutated *IDH* [47,48,49]. *ATRX* mutations are also frequent in patients with low-grade gliomas [50], and they are predictive of a favorable prognosis [51,52,53]. Furthermore, *ATRX* mutations are also significantly associated with *IDH* mutations in glioma patients [51,52,53]. *EGFR* mutations are prevalent in multiple tumors and are often associated with adverse prognostic consequences in glioma patients [54,55]. The inclusion of more patients with *IDH1/2* and *ATRX* mutations in the low-risk group and more patients with *EGFR* mutations in the high-risk group indicates that BM-gene-related risk scores can distinguish glioma patients with different prognoses.

The BM is critical for the normal functioning of the CNS since it is an essential component of the blood–brain barrier (BBB) [56,57,58]. Disruption of the BM structure or any dysfunction can destabilize the BBB, resulting in pathological changes in the CNS. Furthermore, there is evidence that some extracellular matrix components in the BM are capable of inducing the invasion of glioma cells [59]. It has been recently reported [60] that the tumor suppressor miR-1298-3p reduces the proliferative and invasive abilities of glioma cells by downregulating the BM-related gene *NID1*. In addition, studies have shown that aging and stroke can alter the BM composition, which may impair functional recovery in older patients [61].

Our study has some limitations that ought to be considered. First, we failed to obtain other tumor and clinical parameters, such as radiological data, which could provide additional information regarding tumor prognosis. Furthermore, although we used the CGGA database for the independent validation of our nomogram, we still need to conduct prospective clinical trials to validate our findings for future clinical applications. These aspects will be explored in future studies.

## 5. Conclusions

We used five BM-related genes to construct a risk score prognostic model for WHO grade II/III glioma patients, which can provide guidance for clinical decision making.

## Data Availability

Data supporting the findings of our work can be acquired from the respective authors via reasonable request.

## Supplementary Materials

The following supporting information can be downloaded at: https://www.mdpi.com/article/10.3390/genes13101810/s1, Figure S1: Survival analysis of 14 intersecting genes.
