# Supplementary figures and images for: Basement-Membrane-Related Gene Signature Predicts Prognosis in WHO Grade II/III Gliomas
Source: Genes (Basel). 2022 Oct 7;13(10):1810. doi: 10.3390/genes13101810 (PMC9602375; doi:10.3390/genes13101810)

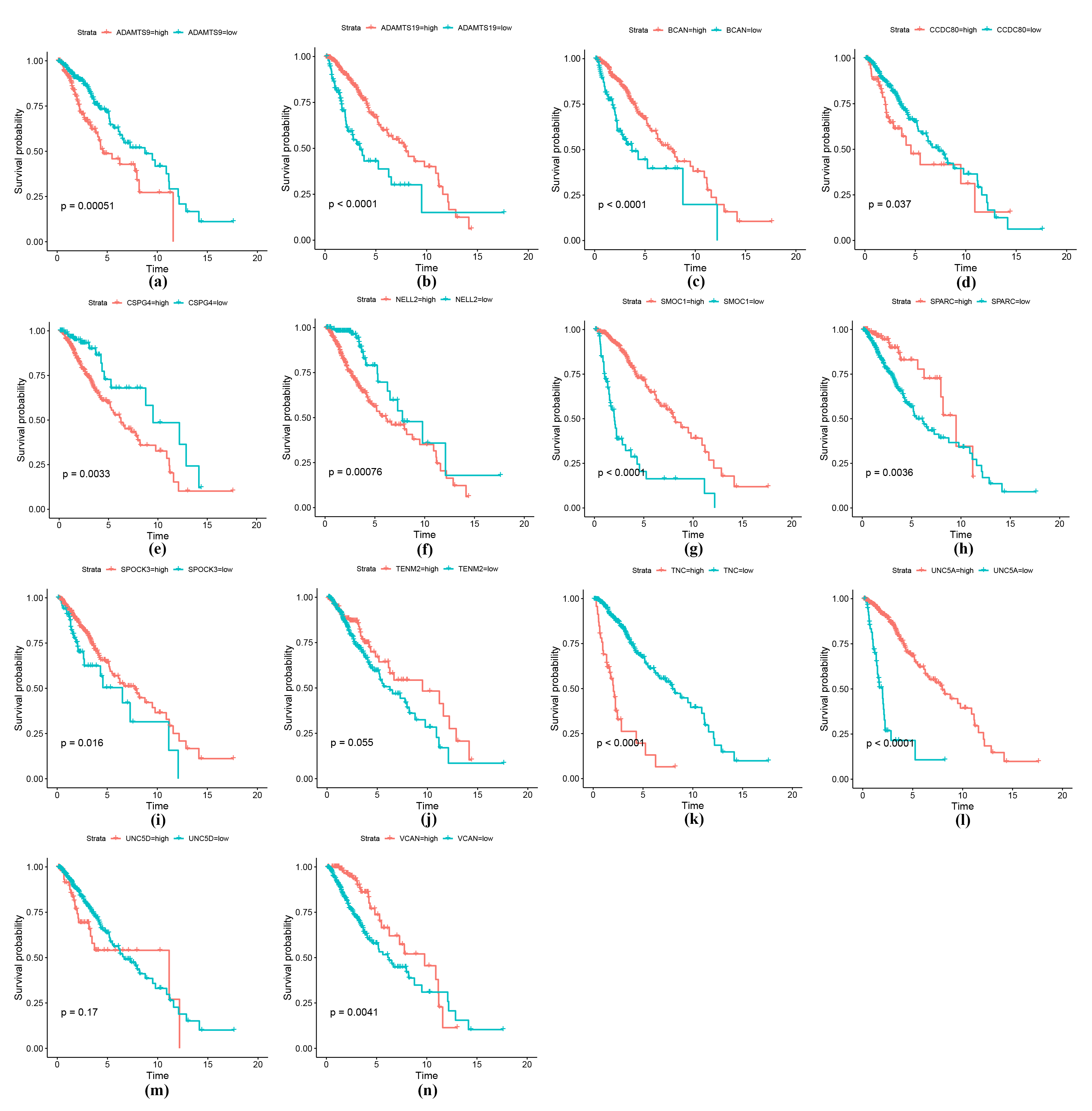

Supplement: Supplementary file 1 [file genes-13-01810-s001.zip › genes-1890938-supplementary.tif]
